# Volume quantification by contrast-enhanced ultrasound and thermodilution: an *in vitro *comparison

**DOI:** 10.1186/cc12138

**Published:** 2013-03-19

**Authors:** IH Herold, G Russo, HC Van Assen, HH Korsten, M Mischi

**Affiliations:** 1 Catharina Ziekenhuis Eindhoven, the Netherlands; 2 University of Technology, Eindhoven, the Netherlands

## Introduction

In clinical practice, blood volumes (BV) are typically measured by thermodilution. Recently, contrast-enhanced ultrasound (CEUS) has been proposed as an alternative minimally invasive approach for BV assessment [1]. This method measures BV using a single peripheral injection of a small bolus of ultrasound contrast agent (UCA) detected by an ultrasound scanner. By measuring the acoustic backscatter, two indicator dilution curves (IDCs) can be derived from two different sites in the circulatory system. IDC analysis permits deriving the mean transit time (MTT) the bolus takes to travel between the injection site and two measurement sites. Assessment of the BV between these sites is obtained by multiplying the difference in MTT (ΔMTT) by the blood flow. In this study, we compare different volumes in an *in vitro *set-up by CEUS with true set-up volumes and thermodilution acquired volumes.

## Methods

The *in vitro *set-up consisted of a centrifugal pump, a network of tubes with variable volumes, an electromagnetic flowmeter to measure and adjust the generated flow, heating devices to maintain constant temperature (37°C), two thermistors for thermodilution measurement, an ultrasound transducer and a pressure stabilizer. A small bolus of UCA diluted in cold saline (1 mg SonoVue^® ^in 20 ml saline at 4°C) was injected into the system. The cold UCA passage through a first and a second region of interest (ROI) was measured simultaneously with the ultrasound transducer and the thermistors. The measurements were performed at different flows and volumes. BVs were estimated using the two different approaches, namely CEUS and thermodilution. The IDCs were processed and fitted separately with a dedicated model to estimate the ΔMTT of the cold UCA bolus between the two ROIs and the two thermistors. All the measurements were repeated three times.

## Results

A linear relation between BVs estimated by the two techniques was observed with a correlation coefficient of 0.94. The bias of CEUS with respect to the true volumes was -40.1 ml; the bias of thermodilution was 84.3 ml. The most prominent differences between the two techniques were observed in case of high volume and low flow, possibly due to different transport kinetics between UCAs and heat.

## Conclusion

Given the good correlation between BVs estimated with CEUS and thermodilution, CEUS is not inferior to thermodilution with the advantage of being minimally invasive.
